# Haplotype Based Association Study between t-PA Gene and Essential Hypertension

**Published:** 2006-06

**Authors:** Kosuke Saito, Tomohiro Nakayama, Naoyuki Sato, Yoshiyuki Kaneko, Ichiro Sato, Akihiko Morita, Teruyuki Takahashi, Masayoshi Soma, Ron Usami

**Affiliations:** 1 *Division of Receptor Biology, Advanced Medical Research Center, Nihon University School of Medicine, Japan;*; 2 *Department of Biotechnology and Applied Chemistry, Toyo University Graduate School of Engineering, Japan;*; 3 *Fifth-year Medical Student, Nihon University School of Medicine, Japan;*; 4 *Department of Obstetrics and Gynecology, Nihon University School of Medicine, Japan;*; 5 *Department of Neurology, Division of Neurology, Department of Medicine, Nihon University School of Medicine, Japan;*; 6 *Department of Neurology, Graduate School of Medicine, Nihon University, Japan;*; 7 *Division of Nephrology and Endocrinology, Department of Medicine Nihon University School of Medicine, Tokyo, Japan*

**Keywords:** tissue plasminogen activator, essential hypertension, haplotype, single nucleotide polymorphism, genetic, association study

## Abstract

Several previous studies have shown that essential hypertension (EH) is associated with fibrinolysis. Tissue plasminogen activator (t-PA) plays a key role in fibrinolysis. Thus, it is possible that the t-PA gene is a susceptibility gene of EH. However, there have been no reported studies of association between EH and the t-PA gene using single nucleotide polymorphisms (SNPs). The aim of the present haplotype-based case-control study was to investigate whether SNPs in the human t-PA gene are associated with EH. We performed a genetic association study using 3 SNPs (rs7007329, rs8178750, rs4471024). The subjects were 276 EH patients and 283 age-matched normotensive (NT) individuals. There were no significant differences in overall distribution of genotypes or alleles between EH patients and NT subjects. Also, there were no significant differences in the haplotype-based case-control study. The present results do not indicate an association between the t-PA gene and EH.

## INTRODUCTION

Essential hypertension (EH) is caused by both genetic and environmental factors. It is generally presumed that EH is a multifactorial inheritance disorder involving several susceptibility genes. Thus, it is important to identify susceptibility genes of EH. However, although there have been studies of relationships between several candidate genes and EH, the major susceptibility genes of EH have not been identified (1).

It was previously predicted that EH is related to disorders in the coagulation-fibrinolytic balance (2, 3). The blood coagulation and fibrinolysis system is regulated by the interaction of many factors. Tissue plasminogen activator (t-PA), which converts plasminogen to plasmin, plays an important role in plasma circulation (4), and increased levels of t-PA antigen may predict risk of cardiovascular diseases such as myocardial infarction (MI) and thrombotic stroke (5, 6). It has been reported that release of t-PA appears to be associated with activity of the endogenous kininogen/kinin system, which is related to EH (7).

Thus, the available evidence suggests that the t-PA gene is associated with EH. However, the relationship between EH and the t-PA gene has not been examined using an association study. The human t-PA gene has been mapped to chromosome 8p12-p11.2. It consists of 14 exons and 13 introns, and spans approximately 32 kb (8). The present haplotype-based case-control study is the first to investigate whether single nucleotide polymorphisms (SNPs) in the human t-PA gene are associated with EH.

## METHODS

### Subjects

The EH subjects were 276 Japanese patients (mean age, 60.0 ± 5.6 years years) with EH diagnosed according to World Health Organization criteria, which included a sitting systolic blood pressure (SBP) of >160 mmHg and/or diastolic blood pressure (DBP) of >100 mmHg on 3 different occasions during the 2 months following the first medical examination. None of the EH subjects was using antihypertensive medication. Patients diagnosed with secondary hypertension were excluded. The controls were 283 Japanese normotensive (NT) healthy individuals (mean age, 77.9 ± 4.6 years). None of the control subjects had a family history of hypertension, and all had a SBP of <130 mmHg and a DBP of <85 mmHg. A family history of hypertension was defined as prior diagnosis of hypertension in a grandparent, uncle, aunt, parent or sibling. Both groups were recruited from the northern area of Tokyo, Japan. Informed consent was obtained from each subject according to a protocol approved by the Human Studies Committee of Nihon University (9).

### Biochemical analysis

Plasma concentrations of total cholesterol and high-density lipoprotein (HDL)-cholesterol and serum concentrations of creatinine and uric acid were measured using the methods of the Clinical Laboratory Department of Nihon University Hospital (10).

### Genotyping

**Figure 1:**
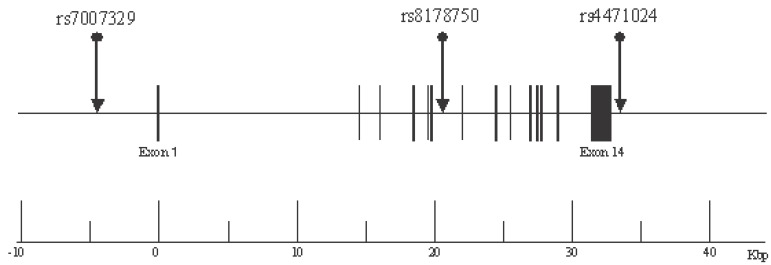
Organization of the human t-PA gene and location of the SNPs used for the association analysis. Closed black boxes indicate exons, and lines indicate introns.

Based on information from the National Center for Biotechnology Information (NCBI) SNP database or the Applied Biosystems (Foster City, CA) - Celera Discovery System (CDS) database (http://www.appliedbiosystems.com), we chose SNPs that had a minor allele frequency of >20%. For the genetic association analysis, we selected the following 3 SNPs in the human t-PA gene: rs7007329 (G/C), located at nucleotide (nt) -4360 upstream of the transcription initiation site; rs8178750 (T/C), nt +20324 in intron 6; rs4471024 (G/A), nt +624 downstream of the terminal codon (Figure 1). Genotypes were determined using Assays-on-Demand kits (Applied Biosystems) and TaqMan PCR, as described previously (11).

### Haplotype-based case-control analysis

We performed haplotype-based case-control analysis of 4 combinations of the 3 SNPs. The frequency of each haplotype was estimated using the expectation/maximization (EM) algorithm (12, 13). Haplotype and linkage disequilibrium (LD) analyses were performed using the software SNPAlyze version 3.2 (DYNACOM Co., Ltd. Yokohama, Japan), which is available from the website http://www.dynacom.co.jp/products/package/snpalyze/index.html.

### Statistical analysis

Data are shown as mean ± SD. Differences in clinical data between the EH and NT groups were assessed using analysis of variance (ANOVA) followed by a Fisher’s protected least significant difference (PLSD) test. Hardy-Weinberg equilibrium was assessed using chi-square analysis. If expected values were small (<2.0), the genotypes with the small expected values were combined (14). The overall distribution of SNP alleles was analyzed using 2 × 2 contingency tables, and differences in distribution of the SNP genotypes between the EH patients and NT controls were assessed using a 2-sided Fisher exact test and multiple logistic regression analysis. Statistical significance was established at *p*<0.05. The threshold value of the frequencies of the haplotypes included in the analysis was set to 1%. All haplotypes below the threshold value were excluded from the analysis. Overall distribution of haplotypes was analyzed using 2 × m contingency tables, with a *p* value of <0.05 considered to indicate statistical significance. The *p* value of each haplotype was determined using chi-square analysis, the permutation method, and SNPAlyze version 3.2 (14). Pair-wise linkage disequilibrium (LD) patterns for the t-PA gene were evaluated using |D’| and r2.

## RESULTS

Table 1 shows clinical characteristics of the subjects. There were significant differences in body mass index (BMI), SBP and DBP between EH and NT subjects. Among male subjects, there were significant differences in pulse rate, total cholesterol, smoking habit, uric acid and alcohol consumption between EH and NT subjects. There were no significant differences in creatinine or HDL-cholesterol between EH and NT subjects.

**Table 1:** Characteristics of study participants

	Total		*p* value	Men		*p* value	Women		*p* value
	EH	NT		EH	NT		EH	NT	

Number of subjects	276	283		180	189		96	94	
Age (years)	51.0 ± 5.6	51.4 ± 8.6	0.517	50.9 ± 5.8	51.9 ± 6.7	0.138	51.2 ± 5.2	50.4 ± 11.5	0.568
BMI (kg/m^2^)	24.6 ± 3.8	22.7 ± 3.3	<0.001	24.7 ± 3.7	22.9 ± 3.2	<0.001	24.3 ± 4.1	22.4 ± 3.4	0.001
SBP (mmHg)	173.9 ± 20.4	112.8 ± 10.6	<0.001	171.8 ± 19.6	113.1 ± 10.3	<0.001	177.8 ± 21.5	112.1 ± 11.4	<0.001
DBP (mmHg)	105.4 ± 13.5	69.6 ± 8.3	<0.001	105.6 ± 13.5	70.3 ± 7.9	<0.001	105.1 ± 13.5	68.2 ± 9.0	<0.001
Pulse (beats/min)	77.7 ± 15.5	73.8 ± 14.1	0.006	77.6 ± 16.3	73.3 ± 15.4	0.022	77.9 ± 14.0	74.8 ± 10.9	0.142
Creatinine (mg/dl)	0.85 ± 0.25	0.83 ± 0.22	0.422	0.94 ± 0.24	0.90 ± 0.20	0.097	0.68 ± 0.18	0.70 ± 0.18	0.394
Total cholesterol (mg/dl)	210.5 ± 36.5	200.7 ± 41.6	0.005	204.9 ± 34.5	196.4 ± 38.6	0.033	220.3 ± 38.1	209.3 ± 46.1	0.078
HDL cholesterol (mg/dl)	57.0 ± 17.7	56.5 ± 17.4	0.753	53.5 ± 16.8	54.3 ± 16.0	0.686	63.0 ± 17.5	61.0 ± 19.3	0.491
Uric acid (mg/dl)	5.7 ± 1.6	5.4 ± 1.5	0.104	6.2 ± 1.5	5.8 ± 1.4	0.032	4.7 ± 1.5	4.6 ± 1.2	0.677
Alcohol consumption (%)	67.7	60.1	0.098	83.7	72.2	0.017	37.5	35.5	0.801
Smoking (%)	54.2	42.0	0.011	64.7	52.8	0.038	33.7	20.6	0.078

BMI, body mass index; SBP, systolic blood pressure; DBP, diastolic blood pressure; HDL, high density lipoprotein; EH, essential hypertension; NT, normotension.

Table 2 shows the distributions of genotype and allelic frequencies for the 3 SNPs in EH patients and NT subjects. There were no significant differences in genotype or allelic distributions of any of the SNPs between the two groups, for total subjects, male subjects or female subjects.

**Table 2:** Genotype and allele distributions in EH patients and NT subjects

		Total		*p* value	Men		*p* value	Female		*p* value
**Number of participants**		276	283		180	189		96	94	
**Variants**		EH	NT		EH	NT		EH	NT	
rs7007329	Genotype									
	G/G	20 (0.072)	18 (0.064)		12 (0.067)	11 (0.058)		8 (0.083)	7 (0.074)	
	G/C	109 (0.395)	115 (0.406)		71 (0.394)	73 (0.386)		38 (0.396)	42 (0.447)	
	C/C	147 (0.533)	150 (0.530)	0.901	97 (0.539)	105 (0.556)	0.919	50 (0.521)	45 (0.479)	0.775
	Allele									
	G	149 (0.270)	151 (0.267)		95 (0.264)	95 (0.251)		54 (0.281)	56 (0.298)	
	C	403 (0.730)	415 (0.733)	0.906	265 (0.736)	283 (0.749)	0.696	138 (0.719)	132 (0.702)	0.721
rs8178750	Genotype									
	T/T	3 (0.011)	1 (0.004)		1 (0.006)	0 (0)		2 (0.021)	1 (0.011)	
	T/C	11 (0.040)	10 (0.035)		6 (0.033)	7 (0.037)		5 (0.052)	3 (0.032)	
	C/C	262 (0.949)	272 (0.961)	0.563	173 (0.961)	182 (0.963)	0.581	89 (0.927)	90 (0.957)	0.614
	Allele									
	T	17 (0.031)	12 (0.021)		8 (0.022)	7 (0.019)		9 (0.047)	5 (0.027)	
	C	535 (0.969)	554 (0.979)	0.313	352 (0.978)	371 (0.981)	0.722	183 (0.953)	183 (0.973)	0.294
rs4471024	Genotype									
	G/G	49 (0.177)	57 (0.201)		29 (0.161)	39 (0.207)		20 (0.208)	18 (0.192)	
	G/A	133 (0.482)	130 (0.459)		88 (0.489)	87 (0.460)		45 (0.469)	43 (0.457)	
	A/A	94 (0.341)	96 (0.339)	0.752	63 (0.350)	63 (0.333)	0.533	31 (0.323)	33 (0.351)	0.908
	Allele									
	G	231 (0.418)	244 (0.431)		146 (0.406)	165 (0.437)		85 (0.443)	79 (0.420)	
	A	321 (0.582)	322 (0.569)	0.670	214 (0.594)	213 (0.563)	0.395	107 (0.557)	109 (0.580)	0.658

EH, essential hypertension; NT, normotension.

There were no significant differences in haplotype distributions between EH patients and NT subjects (Table 3).

**Table 3:** Haplotype distribution analysis using combinations of the 3 SNPs

Combination of SNPs	Overall *P*-value Haplotype	EH	NT	Individual chi-square	Individual *P*-value Individual

rs7007329/rs8178750/rs4471024	0.457CCA	0.534	0.508	0.646	0.422
	GCA	0.027	0.043	1.861	0.173
	CTA	0.020	0.018	0.087	0.768
	CCG	0.179	0.208	1.389	0.239
	GCG	0.241	0.223	0.426	0.514
rs7007329/rs8178750	0.954CC	0.712	0.716	0.020	0.887
	GC	0.267	0.266	0.003	0.957
	CT	0.020	0.018	0.087	0.768
rs7007329/rs4471024	0.344CA	0.553	0.526	0.762	0.383
	GA	0.029	0.043	1.458	0.227
	CG	0.177	0.207	1.532	0.216
	GG	0.241	0.224	0.429	0.512
rs8178750/rs4471024	0.735CA	0.558	0.551	0.055	0.815
	TA	0.024	0.018	0.493	0.483
	CG	0.418	0.431	0.191	0.662

EH, essential hypertension; NT, normotension.

Table 4 shows pair-wise linkage disequilibrium (LD) patterns for the t-PA gene. All SNPs were located in 1 haplotype block.

**Table 4:** Pairwise linkage disequilibrium(LD) of t-PA gene

SNPs	rs7007329	rs8178750	rs4471024

rs7007329	|D´|	0.3452	0.7188
	r^2^	0.0009	0.2481
	rs8178750	|D´|	0.7197
		r^2^	0.0085
		rs4471024	|D´|
			r^2^
|D´|> 0.5		|D´|> 0.3	
r^2^ >0.25			

## DISCUSSIONS

In the present study, using haplotype-based case-control analysis and 3 SNPs, we did not find an association between EH and the human t-PA gene.

There have been several previous studies of relationships between the fibrinolytic system and EH. The fibrinolytic system consists of many factors, including t-PA and plasminogen activator inhibitor 1 (PAI-1). A study by Keskin *et al*. suggests that indicators of fibrinolytic activity such as plasma levels of PAI-1 and beta thromboglobulin (BTG) are associated with EH, but they did not find a relationship between EH and plasma levels of t-PA (15). In addition, the function of t-PA is modulated by interaction with other factors such as PAI-1. Jastrzebska *et al*. studied relationships between t-PA levels and PAI-1 gene polymorphisms in EH and NT subjects. Their comparison of the 4G allele of an insertion/deletion polymorphism in PAI-1 gene carriers (4G/4G homozygotes) revealed that EH patients had significantly higher levels of fibrinogen (*p*=0.045), PAI-1 (*p*=0.009), and t-PA (*p*=0.007) than NT controls (16). Their data indicate that the t-PA gene is associated with atherothrombotic disease, but not EH.

In a previous association study using an Alu-repeat polymorphism in the t-PA gene, there were no significant differences between EH and NT subjects (17). The present strategy of haplotype-based case-control analysis is a more effective method of identifying susceptibility genes than case-control analysis using 1 plolymorphism (18, 19). Our previous LD analysis showed that the |D’| and r2 values of the 3 present SNPs were suitable for haplotype-based case-control analysis (20, 21). Moreover, those 3 SNPs are very useful as genetic markers because they have high minor allele frequencies (>20%).

In conclusion, the present results do not indicate an association between the t-PA gene and EH.
